# Dysregulation of the endothelial nitric oxide pathway is associated with airway inflammation in COPD

**DOI:** 10.1186/s12931-019-1133-8

**Published:** 2019-07-16

**Authors:** Balázs Csoma, András Bikov, Lajos Nagy, Bence Tóth, Tamás Tábi, Gergő Szűcs, Zsolt István Komlósi, Veronika Müller, György Losonczy, Zsófia Lázár

**Affiliations:** 1 Department of Pulmonology, Semmelweis University, Diós árok 1/c, Budapest, 1125 Hungary; 2 NIHR Clinical Research Facility, Manchester University NHS Foundation Trust, Wythenshawe Hospital, Southmoor Road, Manchester, M23 9LT UK; 3 Department of Applied Chemistry, University of Debrecen, Egyetem tér 1, Debrecen, 4032 Hungary; 4 Department of Pharmacodynamics, Semmelweis University, Nagyvárad tér 4, Budapest, 1089 Hungary; 5 Department of Genetics, Cell- and Immunobiology, Semmelweis University, Nagyvárad tér 4, Budapest, 1089 Hungary

**Keywords:** Chronic obstructive pulmonary disease, Nitric oxide, Airway inflammation, Endothelial dysfunction, Cardiovascular comorbidity, Exacerbation

## Abstract

**Background:**

Chronic obstructive pulmonary disease (COPD) is related to endothelial dysfunction and the impaired generation of nitric oxide (NO) from L-arginine by the endothelial NO synthase (eNOS). The relationship between eNOS dysfunctionality and airway inflammation is unknown. We assessed serum asymmetric and symmetric dimethylarginine (ADMA and SDMA) and nitrite/nitrate concentrations, indicators of eNOS function, in patients with COPD and correlated them with markers of inflammation.

**Methods:**

We recruited 15 control smokers, 29 patients with stable and 32 patients with exacerbated COPD requiring hospitalization (20 of them were measured both at admission and discharge). Serum L-arginine, ADMA, SDMA, nitrite and nitrate were measured and correlated with airway inflammatory markers (fractional exhaled nitric oxide concentration - *F*ENO, sputum nitrite and nitrate, sputum cellularity), serum C-reactive protein - CRP, white blood cell count, lung function and blood gases. ANOVA, t-tests and Pearson correlation were used (mean ± SD or geometric mean ± geometric SD for nitrite/nitrate).

**Results:**

Serum L-arginine/ADMA, a marker of substrate availability for eNOS, was lower in stable (214 ± 58, *p* < 0.01) and exacerbated COPD (231 ± 68, *p* < 0.05) than in controls (287 ± 64). The serum concentration of SDMA, a competitor of L-arginine transport, was elevated during an exacerbation (0.78 ± 0.39 μM) compared to stable patients (0.53 ± 0.14 μM, *p* < 0.01) and controls (0.45 ± 0.14 μM, *p* < 0.001). ADMA correlated with blood neutrophil percentage (*r* = 0.36, *p* < 0.01), *F*ENO (*r* = 0.42, *p* < 0.01) and a tendency for positive association was observed to sputum neutrophil count (*r* = 0.33, *p* = 0.07). SDMA correlated with total sputum inflammatory cell count (*r* = 0.61, *p* < 0.01) and sputum neutrophil count (*r* = 0.62, *p* < 0.01). Markers were not related to lung function, blood gases or CRP. L-arginine/ADMA was unchanged, but serum SDMA level decreased (0.57 ± 0.42 μM, *p* < 0.05) after systemic steroid treatment of the exacerbation. Serum nitrite level increased in stable and exacerbated disease (4.11 ± 2.12 and 4.03 ± 1.77 vs. control: 1.61 ± 1.84 μM, both *p* < 0.001).

**Conclusions:**

Our data suggest impaired eNOS function in stable COPD, which is transiently aggravated during an exacerbation and partly reversed by systemic steroid treatment. Serum ADMA and SDMA correlate with airway inflammatory markers implying a possible effect of anti-inflammatory therapy on endothelial dysfunction. Serum nitrite can serve as a compensatory pool for impaired endothelial NO generation.

## Background

The prevalence of cardiovascular comorbidities including ischemic heart disease, heart failure and arrhythmias is higher in patients with chronic obstructive pulmonary disease (COPD) than in the general population [1]. Patients with mild to moderate COPD already present with endothelial dysfunction [2], which is linked with higher arterial stiffness and excess cardiovascular risk [3]. COPD is characterised by airway inflammation, which leads to heightened systemic inflammation [4]. Studies on the association between systemic inflammation and endothelium-dependent vasodilation have provided contradictory findings [5, 6].

Endothelial dysfunction in COPD is related to the altered expression and release of endothelium-derived vasoactive mediators with the key involvement of nitric oxide (NO) [7]. NO is a potent vasodilator, which is produced from L-arginine by the endothelial isoform of the NO synthase (eNOS) [8]. It has a short half-life, and in aqueous solutions it is decomposed to nitrite, while in tissues NO and nitrite are enzymatically oxidized to nitrate [9].

Protein methyltransferases methylate L-arginine residues, which undergo proteolysis to generate asymmetric and symmetric dimethylarginine (ADMA and SDMA) [10, 11]. These amino acids suppress NOS function as ADMA is a competitive inhibitor of the enzyme, while SDMA is a competitor for L-arginine transport. Their roles in the pathomechanism of COPD have recently been studied. Serum ADMA and SDMA but also L-arginine concentrations are increased in COPD and further elevated during an acute exacerbation [12]. Importantly, serum ADMA concentration is an independent risk factor for long-term all-cause mortality in COPD [13], and it is related to increased airway resistance [14]. Nonetheless, the link between the components of the vascular NO pathway and airway inflammation in COPD has not been explored.

Airway inflammation in COPD can non-invasively be studied by the analysis of sputum and exhaled breath. COPD is usually characterized by neutrophilia in sputum and increased level of oxidative and nitrosative stress markers in sputum supernatant [15, 16]. Epithelial and inflammatory cells in COPD airways overexpress inducible NOS (iNOS) [17, 18] to increase NO production and nitrosative inflammation, which can be detected by measuring the exhaled NO concentration [19].

We aimed to compare the serum concentrations of L-arginine, ADMA, SDMA, nitrite and nitrate in patients with stable and exacerbated COPD and smoking control subjects. Measurements were repeated in the convalescence of the exacerbation by hospital discharge. In patients with COPD, we also assessed the associations of these parameters with markers of airway (exhaled NO level, sputum cellularity, sputum nitrite and nitrate concentration) and systemic inflammation (serum C-reactive protein concentration, blood leukocyte counts), lung function variables and blood gas values.

## Methods

### Subjects

Patients with COPD were recruited between March 2016 and December 2017 at the Department of Pulmonology, Semmelweis University, Budapest, Hungary. COPD had previously been diagnosed by a respiratory specialist according to Global Initiative for Chronic Obstructive Lung Disease [20]. Patients presented with stable disease (S-COPD, *N* = 29) or an acute severe exacerbation requiring hospitalization (E-COPD, *N* = 32) with symptoms of recent onset (< 72 h). COPD treatment was unchanged for all patients and they were not treated with systemic steroids or antibiotics in 4 weeks prior to recruitment or hospital admission. Patients with E-COPD did not require ventilatory support, did not have concomitant pneumonia, and their therapy was decided by the treating physician, but all patients received oxygen supplementation, inhaled short-acting bronchodilators and systemic steroid. Control smoking subjects (C) without respiratory symptoms in 4 weeks before inclusion (> 40 years of age, > 10 pack-years) were recruited among employees of the Department (*N* = 15). Subjects were considered ex-smokers if they had stopped smoking at least 6 months before inclusion. All procedures were in accordance with the 1964 Helsinki declaration and its later amendments. The study was approved by the ethics committee and a written informed consent was signed by all participants.

### Study design

Control smokers and patients with S-COPD attended a single visit, while patients with E-COPD were measured within 24 h of hospital admission and when possible also in convalescence (*N* = 20). Cardiovascular co-morbidities (systemic hypertension, heart failure and cerebrovascular accidents) were noted based on medical records and self-report. White blood cell (WBC) count, serum CRP concentration, post-bronchodilator lung function [20] and fractional exhaled nitric oxide (*F*ENO) were measured. Patients filled out the COPD Assessment test (CAT) [21] and blood gases were determined. Serum samples were collected from all subjects and stored at − 80 °C for later analysis. Patients gave spontaneous sputum samples (S-COPD *N* = 13, E-COPD *N* = 17). Furthermore, lung function and *F*ENO measurements were repeated in patients with E-COPD during convalescence (< 24 h before hospital discharge), they filled in the CAT, and second serum (*N* = 19) and sputum (*N* = 9) samples were collected.

### Measurements

#### Routine blood tests

WBC and CRP concentrations were measured in venous blood samples (Sysmex XN-1000, Sysmex Corporation, Kobe, Japan and Beckman Coulter AU680, Beckman Coulter Inc., Indianapolis, IN, USA). Blood gases and pH were determined from arterial samples of patients (S-COPD: *N* = 26, E-COPD: *N* = 30; Cobas b 21, Roche, Switzerland).

#### Lung function tests

Lung function tests were performed according to current guidelines (PDT-111, Piston, Budapest, Hungary) [22, 23]. Spirometry was performed by all subjects. Plethysmography was measured in most patients (S-COPD N = 26, E-COPD *N* = 21).

#### FENO

Fractional exhaled NO concentration was measured at 50 mL/s constant expiratory according to current recommendations (Sievers Nitric Oxide Analyzer i280, GE Analytical Instruments, Boulder, Co, USA) [24]. The mean values of two NO recordings with < 10% difference were used for further calculations. All control subjects, 23 patients with stable COPD and 25 patients at the onset of an exacerbation could perform technically acceptable manoeuvres.

#### Sputum processing

Samples were collected in the morning and processed within 2 h as previously described [15, 25]. Briefly, saliva-free samples were homogenized in 0.1% freshly made dithiothreitol (Sigma-Aldrich, St Louis, MO, USA) and filtered through a nylon mesh. The supernatant was stored at − 80 °C for later analysis. Cells were counted in a haemocytometer, and cell viability was assessed using trypan blue exclusion. Cytospins were prepared and Diff-Quik staining was used to express the differential cell count of non-squamous cells.

#### Determination of serum L-arginine, ADMA and SDMA

The amino acids of the blood serum samples were extracted by solid-phase extraction [26] and then they were derivatized [27]. High performance liquid chromatographic analysis was performed with a Waters 2695 Separations Module equipped with a thermostable autosampler (5 °C) and column module (35 °C). Separation was achieved with a VDSpher PUR C18-M-SE, 5 μm, 150 × 4.6vmm HPLC Column and detected by a Waters 2475 fluorescence detector (Waters, Milford,MA, USA). For the measurements, 10 μl sample was injected and the gradient elution was applied according to Erdelyi-Botor et al. [28]. The detection limit for L-Arginine was 0.1 μmol/L and it was 0.05 μmol/L for both ADMA and SMDA [29].

#### Nitrite and nitrate measurements

The concentrations of nitrite and nitrate were measured in serum samples and sputum supernatants according to an established protocol [30]. In short, proteins in the samples were destroyed by mixing them with 5 volumes of 0.1 M sodium hydroxide solution followed by neutralisation with an equal volume of 0.09 M acetic acid and heating in boiling water-bath for 3 min and centrifugation. The supernatants were analysed by capillary electrophoresis using fused silica capillary and 30 mM sulfate-β-alanine buffer pH 3.8. Nitrate and nitrite were separated using constant − 75 μA current and detected by their UV absorbance at 214 nm. The detection limits for nitrite and nitrate were 0.1 μM and 3 μM in serum and 1 μM and 10 μM in sputum supernatant, respectively.

### Statistical analysis

Demographic data and serum parameters were analysed with ANOVA and post-hoc test, and expressed as mean ± standard deviation, except for serum CRP concentration and packyears, which were compared with the Kruskal-Wallis test and expressed as median (interquartile range). Categorical variables were compared with the chi-square test. The values for *F*ENO, sputum cell count and sputum and serum nitrate and nitrite concentrations were analysed with parametric tests after logarithmic transformation (values of 0 were replaced by 0.01). Outcome parameters were correlated with demographical data, blood results, lung function and blood gas values (Pearson or Spearman correlation). *P* < 0.05 was considered significant (GraphPad Prism 7.0, GraphPad Software, San Diego, USA).

## Results

### Subjects

Patients were older and had more pack-years than control subjects (Table 1). Both COPD groups showed a tendency for a higher prevalence of hypertension cases, and the number of subjects with at least one cardiovascular co-morbidity (hypertension, heart failure or cerebrovascular event) was significantly higher in patients than in smoking controls (control: 33%, S-COPD: 72%, E-COPD: 75%, chi-square test: *p* = 0.01). Patients with E-COPD had an increased blood leukocyte count and elevated serum CRP level compared to stable patients. *F*ENO was higher in patients with E-COPD than in patients with stable COPD or smoking controls. The maintenance inhaled regimens were similar between patients with stable and exacerbated disease. The sputum profiles of patients with stable and exacerbated COPD showed a similar degree of neutrophilic airway inflammation, which did not change at hospital discharge (Table 2).

**Table 1:** Clinical characteristics of patients and controls

	Control	Stable	Exacerbated	*p*-value
		COPD		
Number (male)	15 (6)	29 (13)	32 (21)	(0.15)
Age, years	51 ± 7	63 ± 8***	63 ± 8***	< 0.001
Current/ex-smoker, N	12/3	20/9	19/13	0.36
Pack-years	30 (25–40)	50 (40–75)*	50 (31–78)*	< 0.01
Systemic hypertension, N	5	18	21	0.10
Heart failure, N	0	4	3	0.32
Cerebrovascular event, N	1	2	4	0.70
ICS, N	NA	18	25	0.26
LABA, N	NA	28	29	0.61
LAMA, N	NA	27	29	0.99
Oral theophylline, N	NA	5	11	0.15
White blood cell count, G/L	9.4 ± 3.8	8.6 ± 2.9	11.1 ± 3.9^#^	0.02
CRP, mg/L	4 (2–6)	5 (2–10)	8 (3–14)	0.05
FEV_1_, % predicted	101 ± 14	47 ± 14***	39 ± 13***	< 0.001
FVC, % predicted	109 ± 13	78 ± 19***	65 ± 18***^##^	< 0.001
FEV_1_/FVC	0.78 ± 0.08	0.49 ± 0.09***	0.47 ± 0.09***	< 0.001
RV/TLC	0.31 ± 0.05	0.56 ± 0.12***	0.58 ± 0.10***	< 0.001
Raw, kPa∙s∙L^−1^	0.24 ± 0.08	0.46 ± 0.15***	0.46 ± 0.12***	< 0.001
pH	NA	7.40 ± 0.03	7.41 ± 0.03	0.49
pO_2_, mmHg	NA	60 ± 8	62 ± 10	0.29
pCO_2_, mmHg	NA	43 ± 8	42 ± 7	0.61
CAT score	NA	19 ± 7	21 ± 8	0.16
*F*ENO, ppb	12 ± 2	14 ± 2	26 ± 3**^#^	< 0.01

Data are presented as mean ± SD (geometric mean ± geometric SD for *F*ENO) and compared with ANOVA and post-hoc test or chi-square test (categorical variables) or shown as median (interquartile range) and analysed with Kruskal Wallis and Dunn’s post hoc test. *CAT* COPD Assessment Test, *CRP* C-reactive protein, *ICS* inhaled corticosteroid, *F*ENO fractional exhaled nitric oxide concentration, *FEV*_*1*_ forced expiratory volume in 1 s, *FVC* forced vital capacity, *ICS* inhaled corticosteroid, *LABA* long-acting β2-agonist, *LAMA* long-acting muscarinic antagonist, *N* number, *NA* not applicable, *pCO*_*2*_ partial pressure of carbon dioxide in arterial blood, *pO*_*2*_ partial pressure of oxygen in arterial blood, *Raw* airway resistance, *RV* residual volume, *TLC* total lung capacity. *p < 0.05, **p < 0.01, ***p < 0.001 vs. control, ^#^*p* < 0.05, ^##^*p* < 0.01 vs. stable COPD

**Table 2:** Sputum characteristics in stable and exacerbated COPD

	Stable COPD	Exacerbated COPD	
		Onset	Recovery
Total inflammatory cell count, 10^4^/g sputum	193 (46–773)	202 (61–353)	157 (111–247)
Neutrophils, %	94 (84–95)	85 (70–94)	82 (60–92)
Neutrophils, 10^4^/g sputum	182 (42–657)	196 (52–273)	107 (66–228)
Macrophages, %	5.3 (2.3–12)	9.0 (3.9–298)	8.0 (5.8–18)
Macrophages, 10^4^/g sputum	9.4 (4.7–66)	12 (6.2–46)	13 (6.4–17)
Eosinophils, %	1.5 (0–3.6)	0.8 (0.2–4.6)	0.1 (0–0.5)
Eosinophils, 10^4^/g sputum	2.9 (0–11.8)	1.1 (0.1–3.5)	0.1 (0–3.2)
Lymphocytes, %	0.3 (0–0.8)	0.2 (0–0.7)	0.1 (0–0.8)
Lymphocytes, 10^4^/g sputum	0.2 (0–2.2)	0.1 (0–1.1)	0.1 (0–0.2)

Groups were compared with unpaired t-test (stable vs. exacerbated at onset) or paired t-test (within exacerbation) after logarithmic transformation. Data are shown as median (interquartile range). All *p* values were > 0.10

All patients with E-COPD received systemic corticosteroid treatment during hospitalization, and two-thirds were also treated with antibiotics. At hospital discharge (9 ± 3 days after admission), the CAT symptom score decreased (22 ± 8 vs. 17 ± 9, *p* = 0.03), and FEV_1_ increased compared to the onset of the exacerbation (38 ± 13 vs. 49 ± 19% predicted, *p* = 0.02).

### Serum L-arginine/ADMA and SDMA concentration

**Fig. 1:**
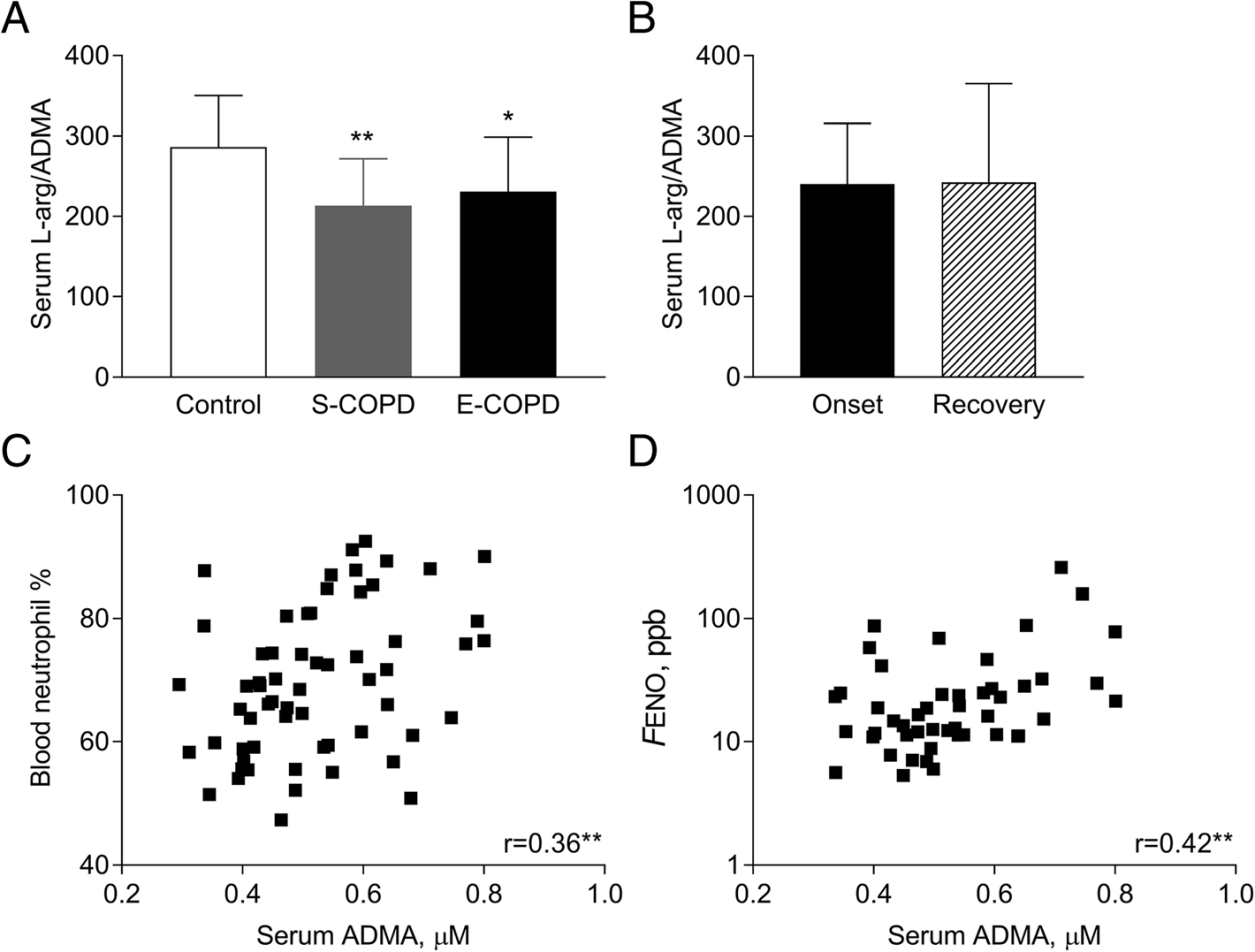
Serum L-arginine/ADMA in smoking controls and patients with COPD. Serum L-arginine/ADMA was analysed among smoking control and patients with stable and exacerbated COPD (**a**, ANOVA with post-hoc test) and between the onset and the recovery of an acute severe exacerbation (**b**, paired t-test). Correlation between serum ADMA concentration and blood neutrophil percentage and *F*ENO was also analysed in patients with stable and exacerbated COPD (**c** and **d**, Pearson correlation). Control: smoking control subjects, S-COPD: stable COPD, E-COPD: exacerbation of COPD, *F*ENO: fractional exhaled nitric oxide concentration. *p < 0.05, **p < 0.01 vs. Control. Data are shown as mean and standard deviation

As ADMA is a competitive inhibitor of NOS, we calculated serum L-arginine/ADMA which indicates L-arginine availability for the enzyme [10]. L-arginine/ADMA was decreased both in stable (*p* < 0.01) and exacerbated COPD (*p* < 0.05) compared to smoking controls (C: 287 ± 64, S: 214 ± 58, E: 231 ± 68; ANOVA *p* < 0.01; Fig. 1 a) and remained suppressed also at the recovery of an acute relapse (243 ± 123; paired t-test, *p* = 0.89, Fig. 1b). In patients with stable and exacerbated COPD, serum L-arginine concentration showed a trend for positive correlation with *F*ENO (*r* = 0.28, *p* = 0.05), but not to other clinical variables or sputum parameters (*p* > 0.1). Of note, serum ADMA correlated with age (*r* = 0.25, *p* = 0.04), blood neutrophil percentage (*r* = 0.36, *p* < 0.01; Fig. 1c) and *F*ENO (*r* = 0.42, *p* < 0.01; Fig. 1d) and a tendency for a direct relationship was observed with sputum neutrophil count (*r* = 0.33, *p* = 0.07).

**Fig. 2:**
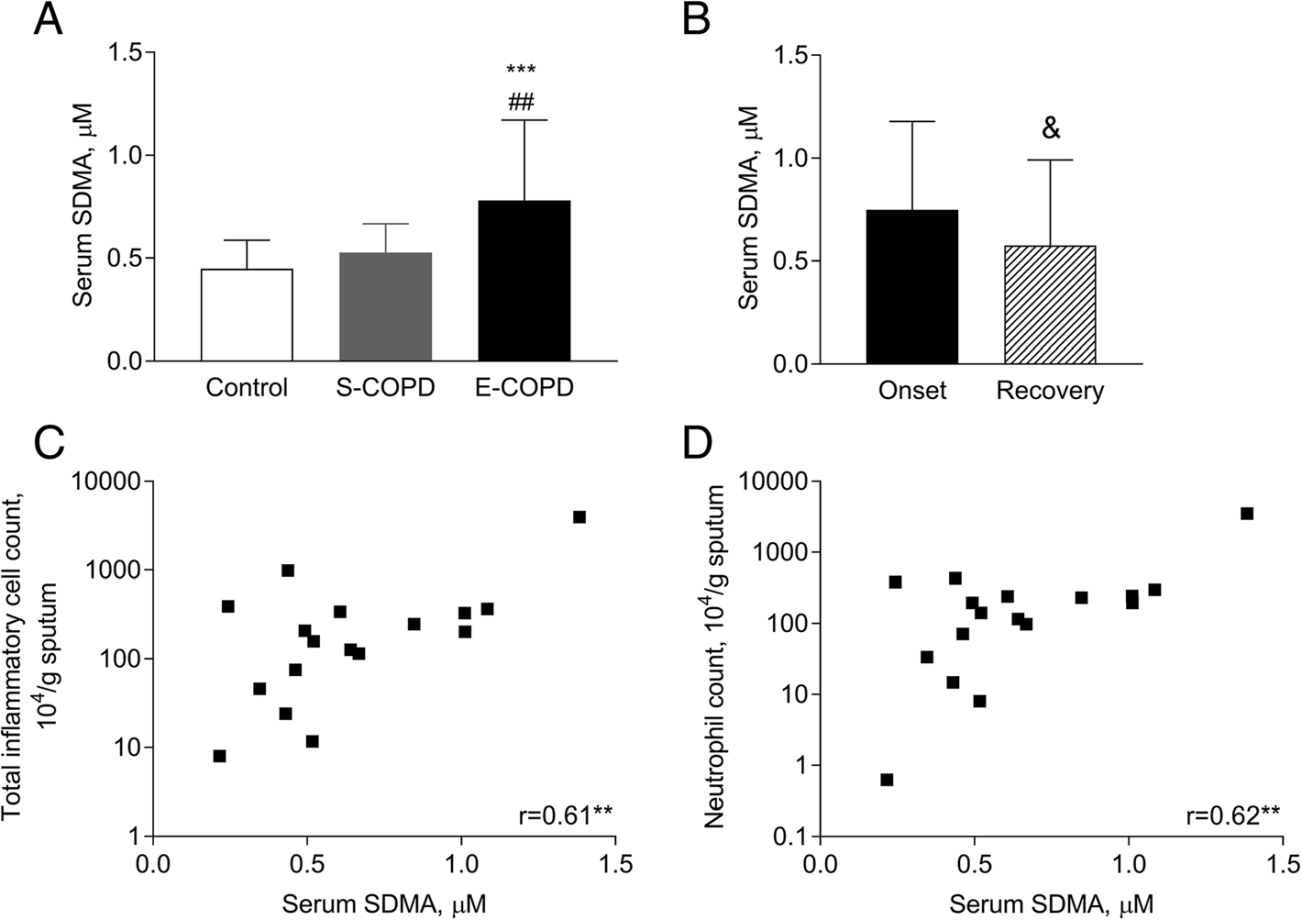
Serum SDMA concentration in smoking controls and patients with COPD. Serum SDMA concentration was compared among smoking controls and patients with stable and exacerbated COPD (**a**, ANOVA) and between the onset and the recovery of an acute severe exacerbation (**b**; paired t-test). Correlation between serum SDMA concentration and sputum inflammatory cell count and neutrophil count was also analysed in patients with E-COPD (**c** and **d**, Pearson correlation). Control: smoking control subjects, S-COPD: stable COPD, E-COPD: exacerbation of COPD. **p < 0.01, ***p < 0.001 vs. Control. ^##^*p* < 0.01 vs. S-COPD, ^&^*p* < 0.05 vs. Onset. Data are shown as mean and standard deviation

Interestingly, the serum concentration of SDMA was elevated only in E-COPD (0.78 ± 0.39 μM vs. C: 0.45 ± 0.14 μM and S-COPD: 0.53 ± 0.14 μM, *p* < 0.001 and *p* < 0.01; Fig. 2a) and it was lowered as a result of treating the exacerbation (0.57 ± 0.42 μM, *p* < 0.05; Fig. 2b). At the onset of an exacerbation, serum SDMA was positively correlated to age (*r* = 0.67, *p* < 0.001), total sputum inflammatory cell count (*r* = 0.61, *p* < 0.01; Fig. 2c) and sputum neutrophil count (*r* = 0.62, *p* < 0.01; Fig. 2d).

### Serum nitrate and nitrite concentration

**Fig. 3:**
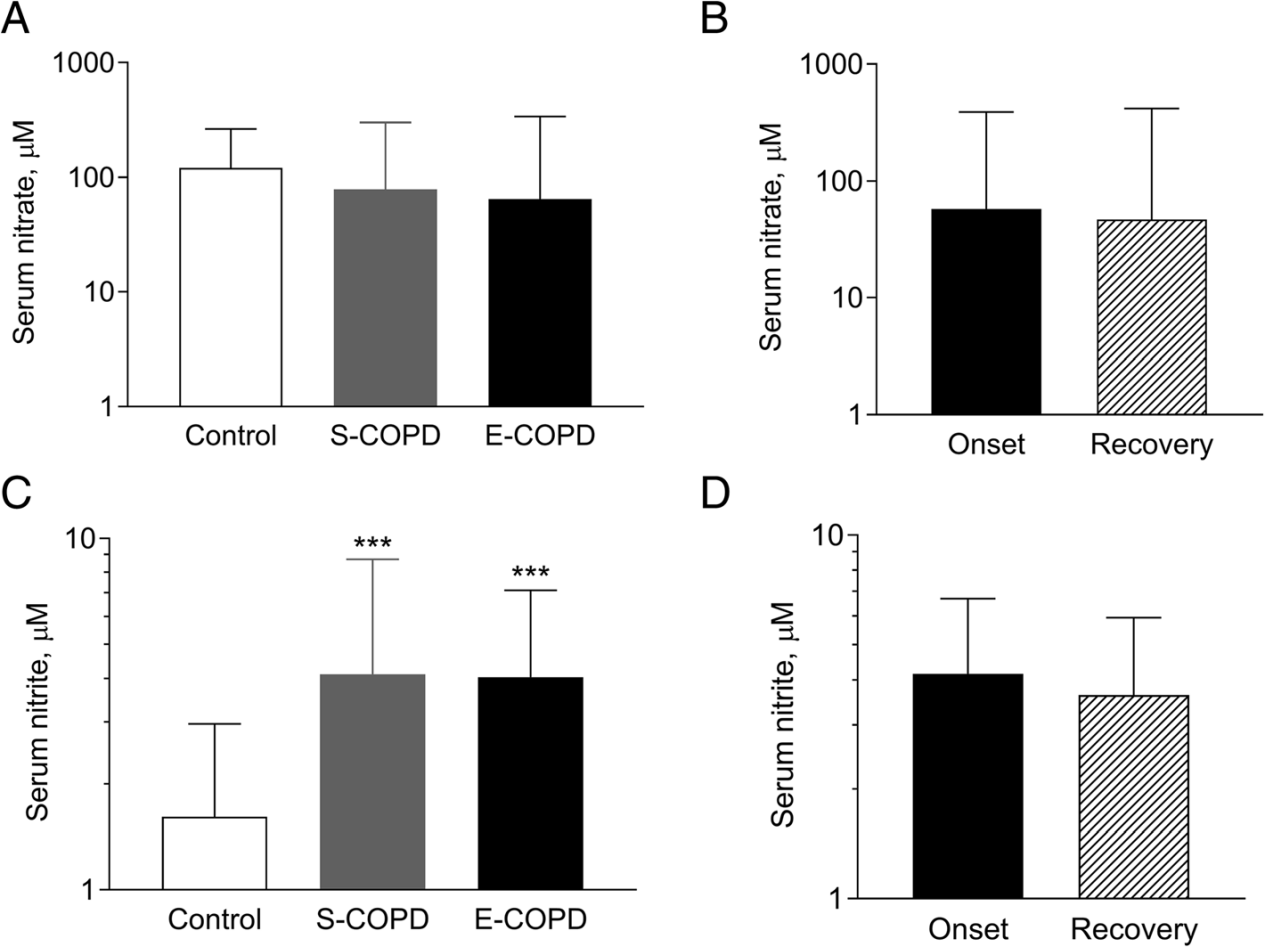
Serum nitrate and nitrite concentration in smoking controls and patients with COPD. Logarithmically transformed serum nitrate and nitrite concentrations were analysed among smoking control and patients with stable and exacerbated COPD (**a** and **c**; ANOVA with post-hoc analysis, ***p < 0.001) and between the onset and the recovery of an acute severe exacerbation (**b** and **d**; paired t-test). Control: smoking control subjects, S-COPD: stable COPD, E-COPD: exacerbation of COPD. Data were analysed after log transformation and are shown as geometric mean and geometric standard deviation

Serum nitrate concentration was similar among smoking control subjects and patients with stable or exacerbated disease (121 ± 2 μM, 79 ± 4 μM, 65 ± 5 μM *p* = 0.36, Fig. 3a), and it showed no difference between the onset and the recovery of an exacerbation (47 ± 9 μM *p* = 0.87, Fig. 3b). Notably, serum nitrite concentration was increased in patients with stable and exacerbated COPD compared to smoking control subjects (4.11 ± 2.12 μM and 4.03 ± 1.77 μM vs. 1.61 ± 1.84 μM, both *p* < 0.001, Fig. 3c), but it showed no difference between hospital admission and discharge in patients with E-COPD (3.64 ± 1.63 μM *p* = 0.26, Fig. 3d).

When data of patients with stable and exacerbated COPD were combined, serum nitrite concentration did not show a correlation with sputum nitrate or nitrite concentrations, *F*ENO, age, pack-years, lung function or blood parameters (*p* > 0.1 for all variables).

### Sputum nitrate and nitrite concentrations in COPD

**Fig. 4:**
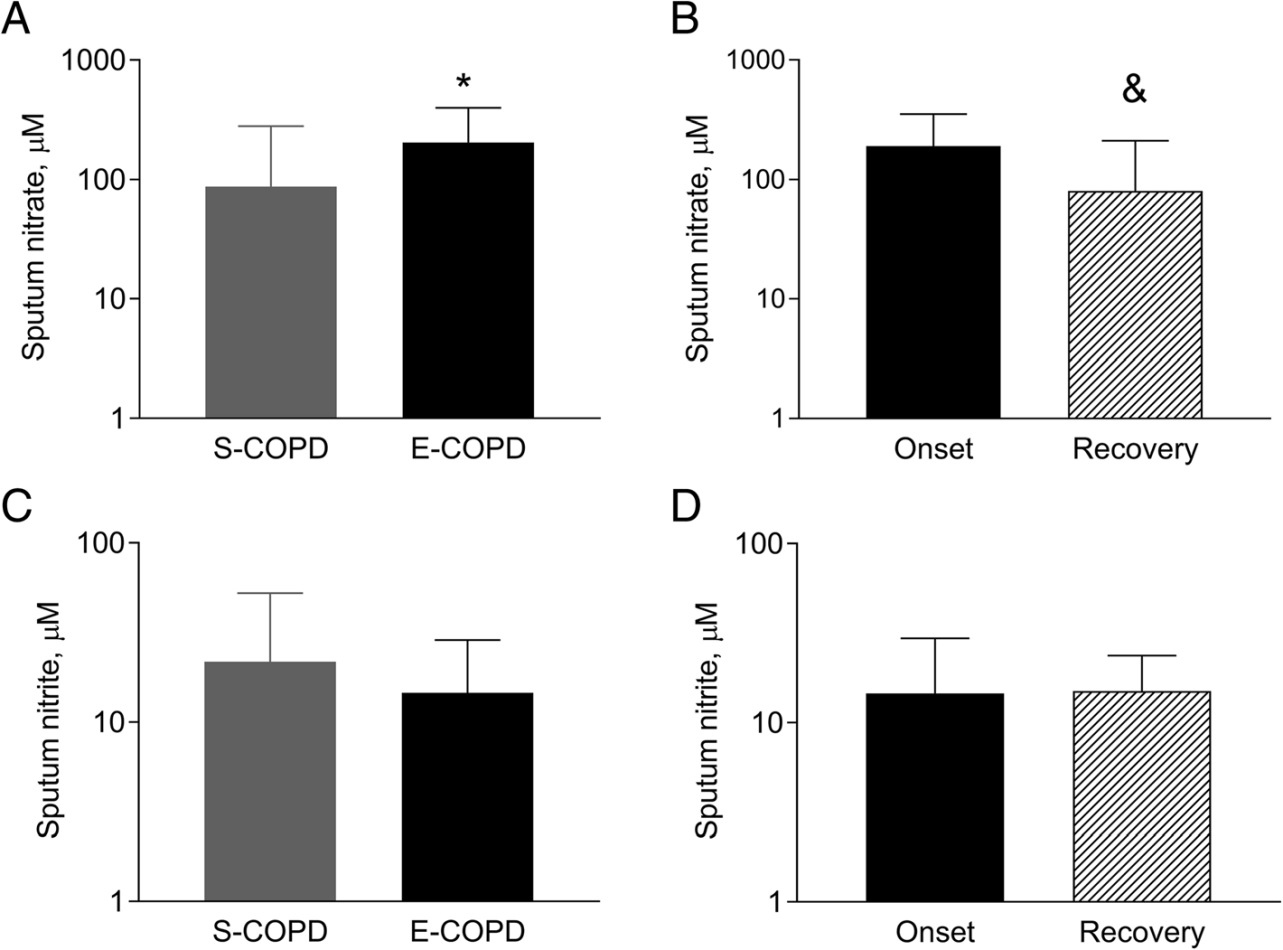
Sputum nitrate and nitrite concentration in COPD. Sputum nitrate and nitrite concentrations were analysed between patients with stable and exacerbated COPD (**a** and **c**; unpaired t-test, *p < 0.05), and between the onset and the recovery of an acute severe exacerbation (**b** and **d**; paired t-test, ^&^*p* = 0.06). S-COPD: stable COPD, E-COPD: exacerbation of COPD. Data were analysed after log transformation and are shown as geometric mean and geometric standard deviation

Sputum nitrate concentration was higher during a relapse than in stable condition (205 ± 2 μM vs. 87 ± 3 μM, *p* < 0.05; Fig. 4a) and was decreased at recovery after adequate treatment (81 ± 3 μM; Fig. 4b). In contrast, sputum nitrite concentration was not changed at an exacerbation compared to the stable state (14.59 ± 1.97 μM vs. 21.74 ± 2.41 μM, *p* = 0.17; Fig. 4c), and no difference was observed between the onset and recovery of a relapse, either (15.02 ± 1.58 μM, *p* = 0.88, Fig. 4d).

## Discussion

Endothelial dysfunction potentially links cardiovascular comorbidities and COPD [1, 31]. In the present study, we found that serum L-Arginine/ADMA ratio is decreased, while serum SDMA level is increased in stable and exacerbated COPD, which can contribute to the impaired vascular NO generation and endothelial dysfunction. Importantly, we showed for the first time that serum ADMA and SDMA concentrations correlate with airway nitrosative stress and neutrophilic inflammation suggesting an association between endothelial dysfunction and airway inflammation in COPD.

The mechanism of vascular wall damage in COPD is not fully understood. Endothelial dysfunction is a component of vascular damage, which has been described as an early signal of increased cardiovascular risk [32]. Nitric oxide generated in the endothelium plays an important role in controlling the vascular tone in COPD. Flow-mediated dilatation (FMD) of the brachial artery, a marker of attenuated NO-mediated vasodilation and endothelial dysfunction, is impaired in patients with COPD [5]. Furthermore, in a cohort of patients with diverse COPD severity and prevalent cardiovascular comorbidities, worse FMD was associated with lower FEV_1_% predicted and lower daily physical activity [6]. The current study contributes to these findings, by showing that impaired endothelial NO signalling relates to airway inflammation.

It was demonstrated that in COPD the biosynthesis of NO is attenuated in the endothelium [7]. Indeed, the serum concentration of the competitive eNOS inhibitor ADMA was shown to be increased in COPD [12]. We extend these findings by proving that serum L-arginine/ADMA ratio, a marker of substrate availability for eNOS, is decreased in stable and exacerbated disease at a similar extent. Importantly, serum ADMA in COPD showed a relationship to systemic (blood neutrophilia) and airway (sputum neutrophilia and exhaled NO) markers of inflammation. Of note, adequate treatment of COPD exacerbation did not modify serum L-arginine/ADMA ratio implying a permanent dysfunction independent of the clinical state of COPD. The mean increase in serum ADMA concentration was 0.1 μM and 0.2 μM in patients with stable and exacerbated COPD, respectively (data not shown), which could already induce circulatory effects [33], and it is in the range associated with an increased risk of cardiovascular death [34].

We measured increased serum concentrations of SDMA in COPD only during an exacerbation, suggesting that eNOS functionality is further and transiently inhibited during a flare-up. In line with this, COPD relapses, especially those requiring hospitalization, convey an increased risk of cardiovascular events [35]. Importantly, serum SDMA was associated with the burden of airway neutrophilia also highlighting a possible link between eNOS dysfunction and airway processes in COPD.

Our findings suggest that drugs targeting airway inflammation might have a beneficial effect on endothelial dysfunction in COPD by increasing eNOS functionality. In support, it was demonstrated that ICS treatment might reduce the risk of acute myocardial infarction in patients with COPD [36], and arterial stiffness can be improved by ICS/LABA therapy [37]. Likewise, we also showed that an increase in serum SDMA concentration during E-COPD is reversed in parallel to a decrease in airway inflammation (reduced sputum nitrate concentration and *F*ENO) as a result of systemic steroid therapy.

We did not find a correlation between ADMA, SDMA and lung function, blood gas parameters or serum CRP. This is in line with the findings of Clarenbach et al., who did not describe an association between endothelial dysfunction and markers of systemic inflammation and oxidative stress, hypoxaemia, age, current smoking and pack-years in patients with stable COPD [6]. However, in our study age correlated with both parameters, which support data from a previous study showing that ADMA and SDMA are involved in endothelial dysfunction in an ageing population [38].

The serum concentration of nitrite was elevated both in stable and exacerbated COPD. Nitrite can serve as a pool for NO as it can be converted into nitric oxide by deoxyhemoglobin-mediated reduction in acidosis or hypoxaemia, which are often present in COPD [39]. In line with this, the generation of nitrite and then NO from dietary supplements rich in inorganic nitrate results in a decrease in systemic blood pressure in patients with COPD [40]. This mechanism can contribute to increasing serum nitrite level in COPD and can presumably result in local NO production in the vasculature to compensate for the attenuated eNOS activity.

Airway activity of the inducible NOS is increased in stable COPD [18], resulting in the increased level of airway nitrite and nitrate [41]. We have previously shown that during an exacerbation the increased nitrosative stress burden can be measured as elevated NO concentration in the exhaled breath [19]. This is extended by the present findings that sputum nitrate concentration is also increased during exacerbation. Serum ADMA concentration in COPD correlated with *F*ENO, but not with sputum nitrate or nitrite levels. This can be explained by the multi-fold reactions of NO in COPD airways including the generation of nitrite/nitrate and peroxynitrite, or its reaction with tyrosine residues.

We cannot exclude that the increased serum nitrite concentration might be related to increased iNOS activity of circulating and vascular cells in COPD and that the altered concentration of serum ADMA and SDMA might also modulate iNOS function on these cells. Blood lymphocytes showed increased iNOS activity in COPD [42], however, neutrophil granulocytes, present in elevated number in patients, did not show iNOS activity in healthy humans [43] with no data available in patients with COPD. In addition, pulmonary arteries showed decreased eNOS, but increased iNOS expression in patients with end-stage disease [44], but it was not confirmed by another study [45].

Our study also has limitations. It would have been relevant to measure endothelial dysfunction using physiological tests and correlate results with serum markers of the NO pathway and their changes during an exacerbation. Furthermore, despite repeated efforts, we could not obtain a sputum sample or a valid exhaled NO measurement in all patients. We did not perform sputum induction and only collected spontaneous samples as sputum induction might convey increased risk during an exacerbation. Samples were obtained from exacerbated patients within 24 h after hospital admission, when all patients received at least one dose of systemic steroid, which could confound the results of the serum and sputum analysis.

## Conclusions

This is the first study to describe a connection between airway inflammation and impaired endothelial NOS activity, a known mechanism involved in endothelial dysfunction in COPD. We found that the substrate availability for eNOS (reflected by L-arginine/ADMA) is decreased in stable and exacerbated COPD, while SDMA is transiently elevated during a relapse. ADMA and SDMA showed correlations to airway inflammatory markers including exhaled nitric concentration, sputum inflammatory and neutrophil cell counts. Our findings confirm impaired eNOS function in COPD and suggest the potential modulation of the vascular NO signalling by the suppression of airway inflammation.

## Data Availability

The datasets used and/or analysed during the current study are available from the corresponding author on reasonable request.

## Abbreviations

ADMA: Asymmetric dimethylarginine
CAT: COPD Assessment Test
COPD: Chronic obstructive pulmonary disease
CRP: C-reactive protein
E-COPD: Exacerbated COPD
eNOS: Endothelial NO synthase
*F*ENO: Fractional exhaled nitric oxide concentration
GOLD: Global Initiative for Chronic Obstructive Lung Disease
ICS: Inhaled corticosteroid
iNOS: Inducible NO synthase
LABA: Long-acting beta_2_-agonist
LAMA: Long-acting muscarinic antagonist
NO: Nitric oxide
S-COPD: Stable COPD
SD: Standard deviation
SDMA: Symmetric dimethylarginine
WBC: White blood cell count
